# Use of Amalgam and Composite Restorations Among 12-Year-Old Children in Israel: A Retrospective Study

**DOI:** 10.3390/biomimetics10120833

**Published:** 2025-12-12

**Authors:** Rimah Nassar, Tali Chackartchi, Haim Doron, Jonathan Mann, Mordechai Findler, Guy Tobias

**Affiliations:** 1 Department of Community Dentistry, School of Dental Medicine, Hebrew University and Hadassah, Jerusalem 91120, Israel; rimah.nassar1@mail.huji.ac.il (R.N.); mannhadassah@gmail.com (J.M.); 2 Department of Periodontology, School of Dental Medicine, Hebrew University and Hadassah, Jerusalem 91120, Israel; tali222@hotmail.com; 3 Research Unit Maccabi-Dent, Tel Aviv 6801298, Israel; haim_d@maccabi-dent.com (H.D.); findlermo@gmail.com (M.F.); 4 Oral Medicine Unit, Sheba Medical Centre Tel-Hashomer, Ramat-Gan 52621, Israel

**Keywords:** amalgam, composite resin, pediatric dentistry, socioeconomic status, dental health reform

## Abstract

Background: This study examined the trends in restorative dental practice among 12-year-old children treated at a nationwide public health maintenance organization in Israel between 2016 and 2022, focusing on the use of amalgam versus composite resin restorations in permanent premolars and molars. Methods: Data were extracted from electronic health records of the second-largest public health organization in Israel, identifying children who underwent restorative treatments during the study period. Restoration rates were compared overall and stratified by gender, socioeconomic status, and number of surfaces restored. Statistical analysis was conducted using SPSS version 27, employing Levene’s test for equality of variances and Welch’s one-way ANOVA. Results: The results showed a statistically significant decline in amalgam use (*p* < 0.05) alongside a marked increase in composite resin restorations (*p* < 0.05), consistent across genders and socioeconomic groups. Notably, composite resins were increasingly selected for complex, multi-surface restorations (*p* < 0.05). Conclusions: These findings highlight a substantial shift in paediatric restorative practice in Israel, reflecting growing preference for composite resins likely influenced by patient demands and national dental reforms that eliminated financial barriers. The observed trend underscores the importance of continued monitoring of material selection to guide evidence-based practice in pediatric dentistry.

## 1. Introduction

Dental caries is the most prevalent disease globally, affecting approximately one-third of the world’s population [1]. As a chronic, multifactorial disease, it involves the progressive demineralization of the dental hard tissues by acids produced from bacterial fermentation of dietary carbohydrates. If untreated, caries can lead to pain, infection, tooth loss, and a significant reduction in quality of life [2]. The burden of the disease extends beyond individual health, representing a considerable challenge for healthcare systems worldwide in terms of treatment costs and prevention strategies [3].

Caries disproportionately impacts socioeconomically disadvantaged children, with higher disease levels observed in low-income populations [4]. Factors such as limited access to dental services, lower oral health literacy, and dietary habits that include frequent consumption of refined sugars contribute to these disparities. Consequently, untreated caries is recognized as both a public health problem and a social justice issue, as it reflects inequalities in access to preventive and restorative care [5].

Community water fluoridation is the most widely implemented public health measure for caries prevention, significantly reducing dental decay in children [6]. Fluoride strengthens enamel by promoting remineralization and inhibiting demineralization, while also reducing the activity of cariogenic bacteria. The effectiveness of water fluoridation has been consistently demonstrated across diverse populations, making it a cornerstone of preventive dentistry [7]. In addition to its protective effect, it is cost-effective, particularly in communities with high levels of caries, thereby contributing to narrowing health inequalities by offering protection to children regardless of their socioeconomic background [8]. Dental amalgam has long been used as a restorative material for direct fillings in posterior teeth [9].

Amalgam Composition: Dental amalgam is an alloy containing roughly half mercury by weight, with the remainder mainly silver, tin, and copper (modern high-copper amalgams often have 10–30% Cu) [10,11]. In the set filling, mercury is largely bound in intermetallic compounds, but small amounts of mercury vapor can be released over time, especially during chewing or teeth grinding. Silver, tin, and copper can also undergo minimal corrosion or dissolution from the amalgam surface, potentially releasing metal ions in trace quantities. These components raise biocompatibility questions: mercury is a known heavy metal toxin at higher exposures, while silver, tin, and copper have their own biological profiles.

### 1.1. Composition of Dental Amalgam and Potential Interactions with Bone

Local Release and Distribution: In the context of a tooth filling, amalgam is usually separated from bone by dentin, enamel, or cementum. Direct interaction with live bone is limited under normal conditions. However, corrosion of amalgam in the moist oral environment can produce metal oxides and sulfides at the restoration margins. Most of these corrosion products remain localized in the tooth or adjacent soft tissues (for example, amalgam tattoos in gingiva caused by embedded amalgam debris), and systemic uptake is low. Mercury vapor released from amalgam can be inhaled and absorbed, disseminating via the bloodstream to various organs, including the kidneys, brain, and potentially bone. Indeed, mercury has an affinity for sulfhydryl groups in proteins and can incorporate into mineralizing tissues; studies have noted that mercury ions may substitute for calcium in hydroxyapatite or carbonate in bone mineral [12]. In practice, the amount of mercury absorbed from typical amalgam fillings is very small—well below occupational safety limits [10], but chronic low-dose exposure is continuous over the restoration’s lifetime. Silver, copper, and tin release from amalgam are even smaller, and these metals tend to either remain bound as surface tarnish or be quickly localized; for example, inorganic tin from diet or materials is known to clear rapidly from blood and only small residues deposit in bone [13]. Overall, the systemic burden of amalgam-derived metals is low, but understanding their potential influence on bone biology requires examining each component.

### 1.2. Biocompatibility of Dental Amalgam with Bone Tissue

Biocompatibility refers to how well a material coexists with living tissues without causing harmful effects. In the case of bone, an ideal biocompatible material might directly bond or integrate with bone or at least not impede the bone’s healing process. Dental amalgam is generally considered biocompatible in that it does not cause systemic illness and has a long track record of safe use [14]. However, amalgam is not bioactive—it does not bond to bone or stimulate bone healing. Instead, when amalgam is in direct contact with bone or connective tissue, the body tends to wall it off with a thin fibrous tissue layer.

Initial Tissue Response: When amalgam fragments or an amalgam filling itself contacts connective tissue or bone, an acute inflammatory response is often observed. Classic animal studies implanted amalgam in rat tissues and noted an early phase (1–2 weeks) of tissue injury characterized by inflammation and even micro-necrosis due to metal ion release [15]. One study found that rat subcutaneous tissue exposed to dental amalgam showed prolonged inflammation, delayed granulation, and disordered collagen formation compared to an inert porcelain implant [16]. This indicates that amalgam’s corrosion byproducts irritate the tissue initially. The inflammation is typically mild to moderate, consisting of macrophages, lymphocytes, and foreign-body giant cells that attempt to isolate the material. Importantly, these reactions diminish with time: by about 4–5 weeks in the rat model, the tissue had adapted and “the implanted amalgam is tolerated” with only minimal chronic inflammation [15]. Essentially, the body forms a capsule around the amalgam, preventing further leaching of irritants into the surrounding bone or soft tissue. This sequence—initial inflammation followed by fibrous encapsulation—is typical of materials that are biocompatible but not biointegrative.

Bone Healing with Amalgam Contact: In dentistry, a relevant scenario is periapical surgery, where a root-end filling material is placed. In the era when amalgam was commonly used for this purpose, clinicians observed that bone healing can occur around amalgam-filled root apices, but often a fibrous scar or encapsulation is present rather than complete regeneration of normal architecture. Newer studies comparing root-end filling materials show that amalgam is outperformed by modern bioceramics in terms of bone healing. For example, mineral trioxide aggregate (MTA) and related cements induce deposition of new hard tissue (cementum/bone) at the resected root tip, whereas amalgam typically does not promote such a regenerative response. The biocompatibility “gap” is one reason MTA has largely replaced amalgam in endodontic surgeries [17]. Nonetheless, healing does occur with amalgam: a successful outcome often shows the periapical radiolucency resolving (bone fill) even if microscopic examination might find a fibrous zone at the amalgam interface. Thus, while amalgam is tolerated in bone, it is considered a passive occupant—bone will generally not bond to it, but rather heal around it. This is acceptable in many cases (the goal is resolution of infection and restoration of bone continuity, which can happen even if a fibrous capsule remains around the material).

One local adverse effect to note is in periodontal health. If an amalgam restoration has an overhanging margin that protrudes into the gum area, it can chronically irritate the adjacent gum and bone. This is not due to chemical toxicity but a mechanical/plaque issue: the overhang traps plaque and fosters inflammation (gingivitis progressing to periodontitis). Studies have documented that teeth with amalgam overhangs show greater loss of adjacent alveolar bone compared to well-contoured fillings [18]. In one study [19], significant loss of alveolar bone height was observed adjacent to amalgam overhangs (*p* < 0.02), independent of the patient’s age. This bone loss is a result of periodontal inflammation caused by the poor restoration contour, not the amalgam material per se—the same would occur with an overhanging composite or crown. Nevertheless, it underscores that amalgam must be properly finished and polished to be biocompatible with periodontal bone; when placed correctly flush with the tooth, amalgam does not induce any bone loss.

In summary, amalgam’s biocompatibility with bone tissue is acceptable but not ideal. It does not integrate with bone, and in direct contact, it causes a transient inflammatory response until a fibrous barrier isolates it. The bone can heal around amalgam (showing the material is essentially inert after initial reactions), but amalgam is not osteoconductive or osteoinductive. Modern dental practice, where possible, avoids leaving large amounts of foreign material in bony sites (for example, amalgam fragments in extraction sockets are removed to prevent tattooing or inflammation). Compared to many other implanted materials, amalgam fares reasonably in biocompatibility tests—it is far from the worst offender (e.g., some polymerizing resins or certain alloys cause more tissue irritation), but it is also not a material that bone loves.

As mentioned, dental amalgam is a mixture of metals (primarily silver, mercury, tin, zinc, and copper) that yields durable restorations able to withstand heavy occlusal forces. Advantages of amalgam include its ease of use, high strength and longevity, and low cost–traits that have made it a preferred material in many low- to middle-income settings [20,21]. However, amalgam lacks aesthetic appeal, conducts heat and cold, and requires the removal of more sound tooth structure during cavity preparation [22].

Amalgam has long been used as a restorative material for direct fillings in posterior teeth [20,21]. As one of the oldest dental restorative materials, its clinical application dates to the early 19th century, and for more than a century, it was considered the “gold standard” in restorative dentistry [22]. Dental amalgam is a mixture of metals (primarily silver, mercury, tin, zinc, and copper) that yields durable restorations able to withstand heavy occlusal forces. Its unique metallurgical properties allow it to expand slightly upon setting, thereby creating a tight marginal seal and reducing microleakage [20].

The advantages of amalgam include its ease of use, high strength and longevity, and relatively low cost–traits that have made it a preferred restorative material in many low- to middle-income settings [12]. Moreover, amalgam performs well in high-load posterior regions, offering clinical survival rates of 10–15 years or more, with some restorations lasting several decades [23]. These characteristics explain its longstanding dominance in restorative dentistry worldwide, particularly in public health services where cost-effectiveness and durability are critical.

However, amalgam also presents several disadvantages [24]. Its metallic color compromises aesthetics, making it unsuitable for anterior teeth and less desirable in populations increasingly concerned with appearance. Amalgam is a thermal conductor, which can result in patient discomfort when exposed to hot or cold stimuli [15]. In addition, amalgam placement often requires removal of more sound tooth structure during cavity preparation to create mechanical retention, which may weaken the tooth in the long term [25].

Beyond clinical considerations, the use of mercury in amalgam has raised concerns regarding occupational exposure for dental professionals, potential environmental pollution, and patient safety. Although extensive scientific evidence supports the safety of amalgam for the general population, growing environmental regulations and global initiatives—including the Minamata Convention on Mercury [26]—have driven efforts to phase down amalgam use in many countries [27]. This international trend, combined with advances in aesthetic restorative materials, has accelerated the shift toward composite resin as the material of choice for posterior restorations [28].

### 1.3. Composite Resin Fillings

Composite resin has been developed over the past several decades as an aesthetic alternative to amalgam, and its mechanical properties have improved enough to permit widespread use in posterior restorations. Composite is composed of a resin matrix (usually acrylic) reinforced with silicate or ceramic filler particles to provide strength and wear resistance [19]. Placing composite restorations requires greater technique-sensitivity and moisture control, and composites are generally less durable than amalgams, with a higher likelihood of replacement due to material wear or secondary caries over time [29,30,31,32]. A notable 2012 study in Brazil [33] found that the choice between amalgam and composite restorative material was primarily determined by clinical factors such as cavity size and surface, rather than patient demographics. In that study, amalgam usage decreased as the number of tooth surfaces involved in the cavity increased—amalgam was mostly chosen for single-surface (occlusal) cavities—and patient-level factors (including socio-demographic variables) did not significantly influence the material choice.

### 1.4. Comparison with Alternative Restorative Materials

Dentistry has trended toward mercury-free alternatives, chiefly resin-based composites, not only for aesthetics but also due to environmental and biological considerations. A comparison of these materials in terms of their impact on bone healing and adjacent bone structures yields the following insights:

Resin Composite Materials avoid the heavy-metal-related concerns associated with amalgam. Indeed, patients with composites will not have mercury in their blood or organs from their fillings. From a bone perspective, composites have not been found to cause any direct negative effects on bone healing. If a composite restoration is well placed, the surrounding bone should remain healthy just as with amalgam. Composites do not corrode or leach metal ions; however, they can leach small amounts of resin monomers or additives, especially if not fully cured. Substances like Bisphenol A glycidyl dimethacrylate (a component in some composites) and its derivatives might have endocrine-disrupting potential. There is ongoing research on whether chronic exposure to trace bisphenol A (BPA) from composites could impact physiology—including bone metabolism, since estrogens influence bone density. High doses of BPA in animal studies have shown some effects on bone architecture [34,35], but the exposure from hardened composite fillings is extremely low and considered insignificant. Epidemiologically, having composite fillings has not been linked to osteoporosis or altered fracture healing. One safety advantage of composites is the absence of galvanic effects; an amalgam next to a gold restoration can create a battery effect (galvanic current), whereas a composite next to metal does not conduct electricity. However, studies implanting amalgam/gold couples in bone found the tiny galvanic currents did not affect bone healing rate [36], so this is more of a transient patient comfort issue (galvanic shock sensation) than a bone health issue. Composites, like amalgam, can cause local issues if misused—for example, a composite overhang can likewise trap plaque and cause periodontal bone loss. Also, if a composite is placed deep under the gums with residual unset resin, it could irritate tissues. But these are clinical technique sensitivities. Overall, composites are considered at least as biocompatible as amalgam, if not more so in certain aspects. They eliminate mercury exposure and, in large studies, have shown no greater systemic health risks [10]. Dentists do note that composites can occasionally cause slight gum or pulp irritation due to monomer release, but serious reactions are very uncommon. In fact, one study found a slightly higher incidence of oral mucosal lesions in patients with many composite restorations compared to amalgam (though still a low incidence). This suggests composites are not entirely inert either, but they do not carry the heavy metal baggage. Importantly, composites have not demonstrated any impairment in bone healing—for instance, when a composite is used as a core under crowns or in restorations near the bone crest, the bone responds normally, provided there is no secondary inflammation from other causes.

That said, all materials must be placed with sound technique; a poorly placed “biocompatible” material can still cause bone loss via secondary factors (plaque, microleakage, etc.). Current clinical practice often prefers these alternatives, especially in young or pregnant patients, to avoid any theoretical mercury risk. From a bone health perspective, this shift is not expected to negatively impact outcomes—if anything, the use of tooth-colored bonded restorations could reduce some mechanical irritation factors and improve periodontal health around restorations when done properly. As research and materials science advance, new restorative materials (e.g., bioactive composites, enhanced ceramics) aim to further improve biocompatibility and even actively aid tissue regeneration, which could one day mean restorations that help surrounding bone stay healthier.

### 1.5. Dental Health Reform

In Israel, a major dental care reform was initiated in 2010 to improve access to pediatric dental services. This reform progressively extended government-subsidized dental care to children, covering preventive and restorative treatments at no cost through the public health funds. By 2019, children and adolescents up to 18 years of age were entitled to free basic dental care as part of the national health basket. Under this program, families incur no out-of-pocket expense for routine check-ups, X-rays, or treatment planning, and only a minimal fixed co-payment (approximately 7$ per treatment) is required for restorative procedures regardless of the filling material used [37,38]. The implementation of this reform led to a marked increase in the utilization of dental services among children, particularly in low-SES communities, and has been credited with improving overall oral health and narrowing socioeconomic disparities in dental disease among Israeli children [38]. Israel had also implemented nationwide community water fluoridation in 2002, achieving broad population coverage and contributing to a decline in caries prevalence. However, legislation in 2014 ceased mandatory water fluoridation, and by the period of this study (2016–2022), no public water supplies were fluoridated.

The aim of this study was to compare the use of amalgam versus composite resin restorations in permanent posterior teeth among 12-year-old children in Israel during the years 2016 through 2022. We specifically examined differences in the utilization of amalgam and composite restorations by patient gender (male vs. female), socio-economic status (SES), year of treatment, and the number of tooth surfaces involved in the restoration.

## 2. Materials and Methods

This retrospective study was based on electronic health records from Maccabi-Dent, the dental service of Maccabi Healthcare Services in Israel, a nationwide public health maintenance organization. Maccabi-Dent is the second-largest provider of dental care in Israel, operating 53 dental clinics and employing over 1100 dentists nationwide. The wide geographic distribution of Maccabi-Dent clinics suggests that its patient population is broadly representative of children across Israel [39]. We utilized the Maccabi-Dent electronic health record database for this study. The study population included all 12-year-old children who received restorative treatments on permanent premolar or molar teeth at Maccabi-Dent clinics between 2016 and 2022. For each included patient, procedural codes were extracted for all posterior restorations performed. We identified codes corresponding to amalgam restorations and composite resin restorations.

Data recorded for each restoration included the patient’s gender, the year of treatment, the number of tooth surfaces restored (categorized as one, two, or three surfaces), and the clinic site. Each patient’s socio-economic status (SES) was determined according to their residential area’s socio-economic cluster as defined by the Israel Central Bureau of Statistics. Socio-economic clusters range from 1 (lowest) to 10 (highest) in the 2015 national index; for this analysis, we grouped clusters 1–3 as low SES, 4–6 as medium SES, and 7–9 as high SES [40]. To calculate restoration rates, the total number of restorations of each type (amalgam or composite) was divided by the number of 12-year-old patients receiving any restorative treatment, for each subgroup (defined by year, clinic, gender, and SES). This “treatment rate” represents the average number of restorations per patient in a given group.

Ethical approval for the study was obtained from the Maccabi Healthcare Services Institutional Review Board (approval MHS-0157-20) and the Maccabi Helsinki Committee. All data were de-identified and analyzed retrospectively.

Data analysis was performed using IBM SPSS Statistics software, version 27.0. Levene’s test was applied to assess the equality of variances for the outcome measures. Because variance homogeneity could not be assumed in all cases, comparisons of amalgam and composite restoration rates were carried out using one-way Welch analysis of variance (ANOVA). We compared restoration rates across the independent variables of interest: year of treatment (2016–2022), patient gender, SES group (low, medium, high), and number of tooth surfaces in the restoration. A significance threshold of *p* < 0.05 was used for all tests.

## 3. Results

A total of 895 patients received at least one amalgam restoration during the study period, compared to 12,661 patients who received composite resin restorations (Table 1). These patients underwent 1535 amalgam restoration procedures and 25,762 composite restoration procedures in total from 2016 to 2022.

**Figure 1:**
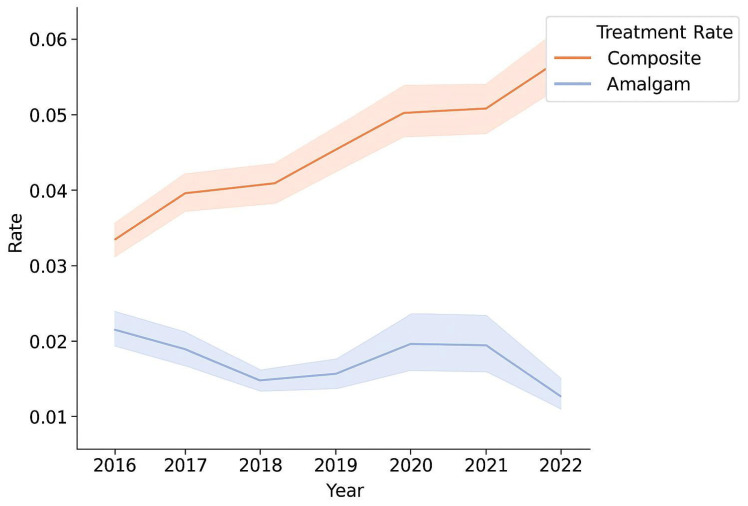
Comparison between the amalgam treatment rate and the composite treatment rate for each year 2016–2022.

Over the 2016–2022 period, there was a clear temporal trend in restorative material choice (Figure 1). The rate of composite resin restorations increased steadily with each year, while the rate of amalgam restorations declined. This inverse trend was statistically significant (*p* < 0.05), indicating a shift away from amalgam toward composite over time.

**Figure 2:**
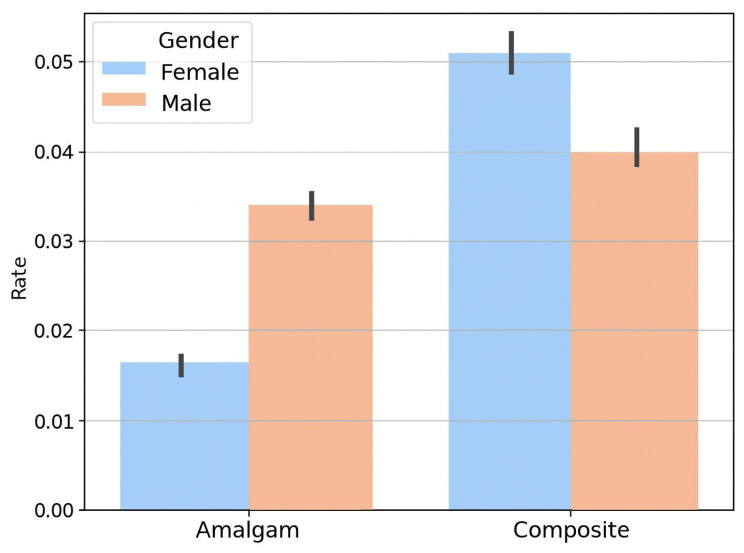
Comparison between the amalgam treatment rate and composite treatment rate for each gender.

When stratified by gender, a significant increase in composite use was observed in both male and female patients (*p* < 0.05 for trend) (Figure 2). By contrast, there was no significant difference between males and females in the rates of amalgam restorations (*p* > 0.05). In each year, composite was increasingly favored in both males and females, and amalgam use remained low in both groups.

**Figure 3:**
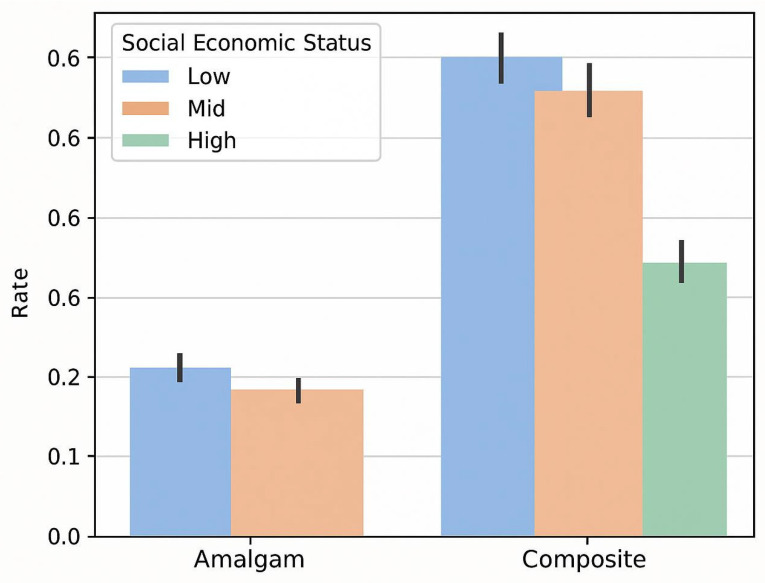
Comparison of amalgam vs. composite restoration rates by socioeconomic status (low, medium, high SES groups).

Stratification by socio-economic status showed that composite resin had largely supplanted dental amalgam as the preferred material across all SES categories (Figure 3). Children from low-SES backgrounds had the highest overall rates of restorative treatments (for both amalgam and composite), but the proportionate shift toward composite was evident in low, medium, and high SES groups alike (*p* < 0.05).

**Figure 4:**
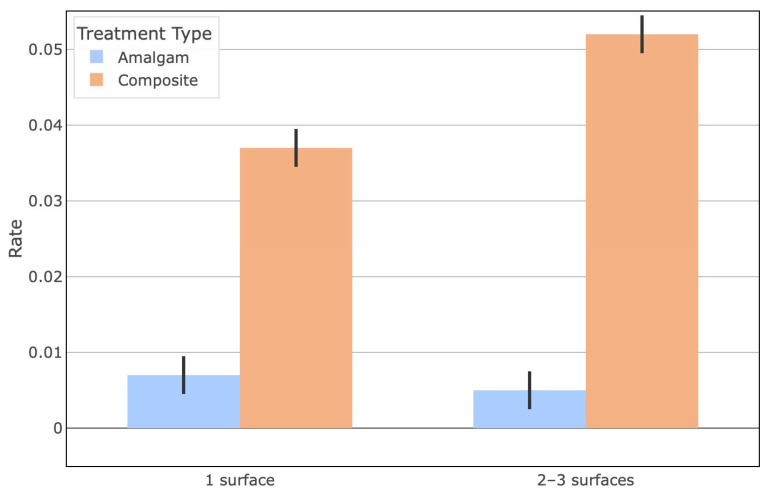
Comparison of amalgam vs. composite restoration rates by number of tooth surfaces involved in the restoration.

Finally, when comparing restorations by the number of tooth surfaces involved, composite resins were significantly more prevalent than amalgam in multi-surface restorations (two- and three-surface cavities) (*p* < 0.05) (Figure 4). There was a noticeable increase in the composite restoration rate for two-surface and three-surface fillings compared to one-surface fillings. In contrast, the amalgam restoration rate did not significantly differ based on cavity size (no significant difference in amalgam rates between one-surface vs. multi-surface cavities, *p* > 0.05). This indicates that composite materials were predominantly used for larger, more complex cavities, whereas amalgam use remained consistently low regardless of the number of surfaces.

## 4. Discussion

The findings of this study indicate a substantial reduction in the use of dental amalgam for 12-year-old patients over the period 2016–2022, alongside a corresponding rise in the use of composite resin restorations. This shift suggests a significant change in clinical practice in Israel away from amalgam and toward composite materials for pediatric restorations. Similar declines in amalgam utilization have been reported internationally; for example, studies in Australia and New Zealand have documented marked decreases in amalgam restorations in recent decades [41,42].

In our analysis, both male and female patients showed a strong move towards composite restorations over amalgams in recent years. There was no significant gender difference in amalgam use, implying that the decline in amalgam was uniform across sexes. With growing awareness of aesthetic dentistry, many patients (and parents) prefer tooth-colored fillings, which may partly explain the increased demand for composites. Consistent with this, a recent study in the United States found that girls were significantly less likely to receive amalgam fillings than boys [43]. Furthermore, an Australian report on dentists’ decision-making noted that non-clinical factors—potentially including patient gender—can influence the choice of restorative material [44]. In our study, although the absolute number of amalgam treatments did not significantly differ between boys and girls, the overall trend for both genders was a clear shift toward composite resin.

Socioeconomic status also appeared to play a role in restorative patterns. We observed that children from lower SES backgrounds underwent a greater number of restorative procedures (of both types) compared to those from higher SES groups. This could reflect higher disease rates or more frequent restoration failures in underprivileged populations. Indeed, previous research has shown that individuals who grow up in lower socioeconomic conditions experience higher failure rates of posterior restorations, necessitating more frequent re-treatment [45,46]. Socioeconomic and behavioral factors have been linked to the longevity of dental restorations and may partly account for the need for additional treatments in lower SES groups [47]. Our findings suggest that when cost is not a barrier—as in Israel’s public dental care system—even lower-SES patients are increasingly receiving composite restorations, reflecting a general preference for aesthetic treatments across the population.

The preference for composite resin over amalgam was especially pronounced for larger cavities in our study. Composite materials were used far more often than amalgam for two-surface and three-surface restorations in 12-year-olds. This trend likely stems from improvements in composite resin technology that allow these materials to successfully restore more extensive cavities with satisfactory longevity. Surveys of pediatric dental practice have indicated that composite resin is the preferred material for Class I and II restorations in children’s permanent [48]. Furthermore, clinical evidence suggests that modern composite resins can perform on par with amalgam even in relatively complex restorations: one five-year study found no significant difference in failure rates between large multi-surface amalgam and composite restorations (approximately 77% success for both materials) [49]. Another analysis noted that as cavity size increases, dentists are increasingly opting for composite, with amalgam now largely reserved for very small restorations [50].

A key strength of this study lies in its use of a large, representative national dataset covering multiple years, which allows for the detection of long-term trends rather than isolated annual fluctuations. By analyzing data across demographic and socioeconomic strata, our methodology ensured that observed changes in restorative patterns reflect genuine shifts in clinical behavior rather than sampling bias. Moreover, the temporal scope of 2016–2022 captures a critical period following major policy reforms, making it possible to correlate systemic changes in healthcare structure with clinical outcomes. This design enhances the robustness of our conclusions and strengthens their relevance to policymakers and dental practitioners alike.

The shift away from amalgam also reflects an evolving understanding of restorative materials in terms of both biocompatibility and environmental sustainability. While amalgam has historically offered mechanical durability, concerns about mercury exposure and waste management have prompted global efforts to phase it down. Composite resins, once considered less durable, have benefitted from substantial advances in filler technology, bonding systems, and polymerization control, resulting in improved wear resistance and marginal integrity. Thus, the clinical preference for composite restorations seen in this study is consistent with evidence-based trends that favor materials combining functionality with safety and aesthetics.

Nevertheless, this study has certain limitations. As a retrospective analysis, it cannot fully capture clinical decision-making rationales, such as a dentist’s experience, parental preference, or specific cavity characteristics. Future studies could complement these findings through prospective designs or qualitative surveys to better understand the behavioral and educational aspects influencing material selection. Additionally, longitudinal follow-up studies on restoration survival rates would help validate whether the increased reliance on composites yields equivalent or improved long-term outcomes compared to amalgam. Such extensions would further strengthen the scientific rigor and policy relevance of this research.

The expansion of Israel’s national health insurance package to include restorative dental treatments for children has been transformative in reducing inequities in oral healthcare. By removing the cost differential between amalgam and composite resin restorations, the reforms ensured that clinical decision-making could be guided primarily by professional judgment and patient preference rather than financial constraints. This shift allowed children from all socioeconomic backgrounds to access the same quality of restorative care, a critical step toward reducing disparities in oral health outcomes.

Importantly, the equalization of costs between amalgam and composite resins appears to have accelerated the adoption of composite as the material of choice. In healthcare systems where financial barriers persist, amalgam often remains the default option due to its lower cost, despite its aesthetic disadvantages and broader concerns regarding mercury content [9]. In contrast, the Israeli reform [24,25] effectively removed this structural bias, empowering families to prioritize aesthetics and minimally invasive treatment, and enabling dentists to align treatment decisions with international best practices.

Beyond individual patient outcomes, these reforms have broader implications for public health and policy. By facilitating greater use of composite resins, the system supports a more patient-centered model of care, one that recognizes the psychosocial value of dental aesthetics for children and adolescents. Additionally, as amalgam use continues to decline globally in accordance with environmental and health policy recommendations (e.g., the Minamata Convention on Mercury [16]), Israel’s experience underscores how financial reforms can align national practice patterns with international public health priorities.

## 5. Conclusions

Our findings highlight a significant shift in restorative practices among 12-year-old children in Israel, with composite resin increasingly replacing amalgam as the preferred. This transition reflects not only the removal of financial barriers within the Israeli healthcare system, where both materials are equally accessible, but also the growing emphasis on patient and provider preferences for improved aesthetics and minimally invasive care. The decline in amalgam use mirrors international trends and underscores the importance of continued surveillance of restorative material utilization. Monitoring these patterns is critical for anticipating long-term oral health outcomes, guiding evidence-based decision making, and informing dental public health policy in Israel and beyond.

## Figures and Tables

**Table 1:** Distribution of Patients and Treatments According to Restoration Material (Amalgam vs. Composite).

Variable	Female N (%)	Male N (%)	Total
Number of patients—Amalgam	476	419	895
Number of patients—Composite resin	7255	5406	12,661
Number of treatments—Amalgam	816	719	1535
Number of treatments—Composite resin	15,078	10,684	25,762

## Data Availability

Research data is available upon request.

## Abbreviations

The following abbreviations are used in this manuscript:

SES	Socio Economic Status
HMO	Health Maintenance Organization
